# Changes in pro-inflammatory cytokines and antimicrobial proteins in elderly women with iron deficiency anemia

**DOI:** 10.12669/pjms.35.2.520

**Published:** 2019

**Authors:** Sinasi Askar, Seyma Nur Deveboynu, Hilal ER, Tunay Kontas Askar, Adnan Adil Hismiogullari

**Affiliations:** 1 *Sinasi Askar Assistant Professor, Department of Nutrition and Dietetics, Cankiri Karatekin University, Cankiri, Turkey*; 2 *Seyma Nur Deveboynu Res Assistant, Department of Nutrition and Dietetics, Cankiri Karatekin University, Cankiri, Turkey*; 3 *Hilal ER Res Assistant, Department of Nutrition and Dietetics, Cankiri Karatekin University, Cankiri, Turkey*; 4 *Prof. Dr. Tunay Kontas Askar Department of Nutrition and Dietetics, Cankiri Karatekin University, Cankiri, Turkey*; 5 *Adnan Adil Hismiogullari Associate Professor, Tibbi Biyokimya Bolumu, Tip Fakultesi, Balikesir Universitesi, Balikesir, Turkey*

**Keywords:** Antimicrobial protein, Cytokines, Iron deficiency anemia

## Abstract

**Objective:**

Iron Deficiency Anemia (IDA) is common nutritional deficiency particularly in women and also common during the older age. Aging increases plasma levels of inflammatory mediators such as pro-inflammatory cytokines and acute phase proteins. However, there is little information about changes in antimicrobial proteins in elderly women with IDA. In this study, we aimed to determine the changes in pro-inflammatory cytokines and newly discovered antimicrobial proteins like hepcidin, chemerin and defensin in elderly women with IDA.

**Methods:**

Blood samples taken from healthy 20 women (55±7 years old) and 20 women with iron deficiency anemia (58±6) were used as material of the study that came to the Cankiri State Hospital in 2016 and 2017 years.

**Results:**

In the study, plasma C-Reactive Protein (CRP) (P <0.01), tumour necrosis factor-α (TNF-α) (P <0.05) and interleukin-6 (IL-6) levels (P <0.05) were significantly increased in group with IDA when compared with healthy group. Also plasma hepcidin (P <0.001), chemerin (P <0.05) and defensin levels (P <0.05) were determined significantly higher than the healthy group.

**Conclusion:**

We found that inflammatory changes occur in elderly women with IDA. Besides pro-inflammatory cytokine levels (CRP, IL-6, TNF-α), antimicrobial protein levels (hepcidin, chemerin, defensin) were found higher in elderly women with IDA because of inflammatory changes.

## INTRODUCTION

Aging is an inevitable process that is affected by genetic, lifestyle and environment. However, the underlying mechanisms of the aging process are not fully understood. It has been shown that aging is closely associated with an increase in pro-inflammatory cytokines.^1^

Iron is an important micronutrient that has a vital role in body. Daily dietary iron requirements are about 1 mg for males and 2-3 mg for adolescences and females. Intake of iron is about 15 mg in daily with diet and only %5-10 of this amount is absorbed in bowel. Iron deficiency anemia (IDA) is caused by problems in the synthesis of hemoglobin. Diagnosis of iron deficiency anemia is that hemoglobin of less than 11.5 g/dl in women and less than 13.5 g/dl in women. This case is a quite common worldwide. Being elderly is a risk factor for development of IDA because of intake drug, appetite changes and insufficient absorption from the bowel.^2^

Cytokines play a major role in the regulation of inflammatory response. The reason for the increased frequency of anemia in old age may be dysregulation of pro-inflammatory cytokines (CRP, TNF-α, IL-6 etc.) with aging. It has been suggested that increased pro-inflammatory cytokines direct or indirect inhibit erythropoietin and thus may lead to anemia.^3^

Also, antimicrobial peptides (AMPs) and proteins play an important role in human immune defence. AMPs are key elements of innate immunity and can possess functions such as apoptosis, wound healing, etc. Defensin, hepcidin and chemerin are some of the antimicrobial proteins.^4^

Hepcidin is a recently discovered hormone which is synthesized in the liver and is an antimicrobial protein displaying antibacterial and antifungal properties and maintains iron balance in the microorganism thus regulating its metabolism. When iron stores are adequate hepicidin production is increased in the liver and erythropoietic signals lead to the reduction in its synthesis. Therefore, it can regulate the way iron is transported from enterocytes to the plasma in the small intestine. During anemia, hepcidin creates an important link between the body’s defense, inflammation, and iron metabolism. A previous study has shown that during infection and inflammation, hepcidin synthesis is considerablely increased owing to stimulation by IL-6.^5^

Chemerin is a recently discovered antimicrobial protein and adipokine. Chemerin is a protein playing a role in immune cell migration, adipogenesis, osteoblastogenesis, glucose homeostasis, etc. It is abundantly in human epidermis to ensure barrier.^6^ Lehrke et al. (2009) showed that chemerin is strongly associated with inflammation parameters.^7^

Defensin is a peptide that has an antimicrobial activity. Defensins contribute to host defence in the small intestine, skin and elsewhere.^8^ Defensins are divided 3 groups as α-defensins, β-defensins, and θ-defensins. Only α- and β-defensins are available in humans.^9^ The human β-defensin gene is the member of the human defensin family that is locally inducible by inflammation.^10^ Reduced expression of defensins compromises host immunity and thus may change the balance toward inflammation.^11^

Although immune changes in anemia has been subjected of many investigations,^3^,^12^ but there is little information about changes in antimicrobial proteins (chemerin, defensin etc.) in elderly women with IDA. Therefore in this study we aimed to determine the changes in pro-inflammatory cytokines and newly discovered antimicrobial proteins like hepcidin, chemerin and defensin in elderly women with IDA.

## METHODS

Blood samples taken from healthy 20 women (55±7 years old) (control group) and 20 women with iron deficiency anemia (58±6) were used as material of the study that came to the Cankiri State Hospital in 2016 and 2017 years. The exclusion criteria included women suffering from inflammatory diseases, women with a known chronic disease and who had taken antibiotics in the last 4 weeks. This study was approved by the Ethics Committee from Kirikkale University School of Medicine. Iron deficiency anemia group was composed of women who had hemoglobin (Hb) level of < 11.5 g/dL, plasma iron < 30 mg/dL and ferritin < 15 ng/mL. Three mL of venous blood was taken from the women and placed in tubes containing anticoagulant and the plasma was separated for biochemical analysis. Plasma samples were placed into the eppendorf tube and stored at -80°C, until analyses.

### Laboratory Methods

Plasma iron, ferritin and hemoglobin levels were performed on an automated analyzer (Roche, DP Moduler System, Tokyo, Japan) using commercial test kits. The procedures for determining the hormone levels were performed as recommended in the relevant catalogues, using micropleyt reader (μQuant Elisa reader, Bio-Tek, USA).

### Pro-inflammatory Cytokine and Antimicrobial Protein Analysis

Plasma levels of TNF-α, IL-6, CRP, defensin and chemerin were measured by ELISA using a commercially available kit (Coat-A-Count kit, YH Biosearch Laboratory, Shanghai Pudong New Area JinHu). Hepcidin (DRG International Inc., USA) levels were measured by enzyme linked immunosorbent assay (ELISA) using a commercially available kit (Phoenix Pharmaceuticals Inc., USA).

### Statistical Analysis

The data obtained in this study was evaluated using a statistical computer package (SPSS 13 for Windows standard version). Data averages were expressed as ± standard error. The normality tests of the data was subjected to statistical differences between groups, by a one way analysis of variance (ANOVA), and the Duncan test as a post-test was applied.

## RESULTS

In this study, plasma CRP (P <0.01), TNF-α (P <0.05) and IL-6 levels (P <0.05) were significantly increased in group with IDA when compared with healthy group. Also plasma hepcidin (P <0.001), chemerin (P <0.05) and defensin levels (P <0.05) were determined significantly higher than the healthy group (Table-I).

**Table-I:** Changes in pro-inflammatory cytokines and antimicrobial proteins profile between the control group and group with IDA.

Parameters	Healthy Controls n=20	Group with IDA n=20
CRP (mg/L)	1.5±0.6^a^	3.4±1.5^b^
TNF-α (pg/mL)	36.45± 12.4^a^	58.21±17.9^b^
IL-6 (pg/mL)	20.72±10.6^a^	39.55±12.1^b^
Defensin (ng/mL)	10.5±2.6^a^	21.2±3.5^b^
Hepcidin (ng/mL)	165,5±50.8^a^	246.70±46.1^b^
Chemerin	89.5±25.4^a^	111.5±31.8^b^

a, b signs indicate statistical differences among the groups (p<0.05).

## DISCUSSION

Aging is an unavoidable process that is affected by genetic, lifestyle and environmental factors. Although, the underlying mechanisms of the aging process are not fully understood.^1^ Aging is associated with inflammation and biomarkers such as IL-6.^13^ Aging process might be a factor in development and progress of anemia with dysregulation of some proinflammatory cytokines.^3^ These studies about changes of antimicrobial proteins throughout Aging and in iron deficiency are not sufficient.

In this study, we demonstrated that antimicrobial proteins such as hepcidin, chemerin, defensin, pro-inflammatory cytokines such as TNF- α and IL-6 and acute phase proteins such as CRP elevated in elderly women with IDA.

Penninx et al. (2004) have found that persons with iron anemia have significantly higher serum levels of CRP, IL-6, and TNF-α.^14^ Gonzalo-calvo et al. (2010) showed that TNF-α is an indicator of aging and its levels increases in elder people.^15^ Also, Roubenoff et al. (1998) showed that production of IL-6 was higher in the elderly subjects than the young subjects. The increased IL-6 correlated with increased CRP, a marker of inflammation.^16^ In this study, we demonstrated that TNF- α, IL-6 and CRP increased in elderly women with IDA.

Antimicrobial proteins play an important role in human host defense. Defensin, chemerin and hepcidin are among antimicrobial proteins.^4^ Especially hepcidin that among these hormones, is an peptide associated with iron metabolism. The synthesis of hepcidin is widely stimulated by inflammation.^17^ IL-6 parameter that can be used as markers of inflammatory processes increases with aging.^15^ Because the inflammation increases with aging, hepcidin also increases.^18^ In our study, plasma hepcidin levels in the control group were significantly lower than IDA group.

Defensin is peptide that has an antimicrobial activity and contributes to host defence.^8^ β-defensins are also capable of inhibiting inflammation. Human defensins are induced on exposure to bacterial infection, proinflammatory stimuli, etc.^19^ TNF-α induces defensin expression in keratinocytes but the mechanism is unclearly.^20^ In this study, plasma defensin levels in iron deficiency anemia group were significantly higher than the control group.

Chemerin plays role as a broad spectrum antimicrobial protein in host defense.^6^ Lehrke et al. (2009) showed that chemerin is strongly associated with inflammation parameters.^7^ Chemerin levels were correlated with CRP, TNF-α and IL-6 concentrations.^7^,^21^ Furthermore, TNF-α induces chemerin synthesis and secretion by adipocyte.^22^ There are the studies that showed which chemerin levels increased with age.^23^,^24^ Zylla et al (2017) have shown chemerin levels are higher in women than men.^23^ In this study, plasma chemerin levels in iron deficiency anemia group were significantly higher than the control group.

## CONCLUSIONS

We found that inflammatory changes occur in elderly women with IDA. Besides pro-inflammatory cytokine levels (CRP, IL-6, TNF-α), antimicrobial protein levels (hepcidin, chemerin, defensin) were found higher in elderly women with IDA because of inflammatory changes.

### Author`s Contribution

**SA:** Conceived, designed and did statistical analysis.

**SND:** Did data collection and manuscript writing.

**HE:** Did data collection and manuscript writing.

**TKA:** Did review and final approval of manuscript.

**AAH:** Editing of manuscript.
